# RNA Interference: A Novel Source of Resistance to Combat Plant Parasitic Nematodes

**DOI:** 10.3389/fpls.2017.00834

**Published:** 2017-05-19

**Authors:** Sagar Banerjee, Anamika Banerjee, Sarvajeet S. Gill, Om P. Gupta, Anil Dahuja, Pradeep K. Jain, Anil Sirohi

**Affiliations:** ^1^Division of Nematology, Indian Agricultural Research Institute (ICAR)New Delhi, India; ^2^Centre for Biotechnology, Maharshi Dayanand UniversityRohtak, India; ^3^Division of Biochemistry, Indian Agricultural Research Institute (ICAR)New Delhi, India; ^4^National Research Centre on Plant Biotechnology (ICAR)New Delhi, India

**Keywords:** plant parasitic nematodes, host delivered RNAi, root-knot nematodes, cyst nematodes, dsRNA, siRNA

## Abstract

Plant parasitic nematodes cause severe damage and yield loss in major crops all over the world. Available control strategies include use of insecticides/nematicides but these have proved detrimental to the environment, while other strategies like crop rotation and resistant cultivars have serious limitations. This scenario provides an opportunity for the utilization of technological advances like RNA interference (RNAi) to engineer resistance against these devastating parasites. First demonstrated in the model free living nematode, *Caenorhabtidis elegans*; the phenomenon of RNAi has been successfully used to suppress essential genes of plant parasitic nematodes involved in parasitism, nematode development and mRNA metabolism. Synthetic neurotransmitants mixed with dsRNA solutions are used for *in vitro* RNAi in plant parasitic nematodes with significant success. However, host delivered *in planta* RNAi has proved to be a pioneering phenomenon to deliver dsRNAs to feeding nematodes and silence the target genes to achieve resistance. Highly enriched genomic databases are exploited to limit off target effects and ensure sequence specific silencing. Technological advances like gene stacking and use of nematode inducible and tissue specific promoters can further enhance the utility of RNAi based transgenics against plant parasitic nematodes.

## Introduction

Plant parasitic nematodes (PPNs) have emerged as a severe threat to crop production and are responsible for an estimated loss of US $173 billion annually to world agriculture (Elling, 2013). In addition to the direct damage caused to the plants, nematode infection facilitates subsequent attack by other plant pathogens such as bacteria and fungi. Sedentary endoparasites are the most significant and economically damaging PPNs which include the genera *Meloidogyne* [root-knot nematodes (RKNs)], *Heterodera* and *Globodera* [Cyst Nematodes (CNs)]. The RKNs have a broad host range and form characteristic root galls on the host roots, while CNs have a comparatively constricted host range. The life cycles of these parasites are characteristically different from each other involving complex interactions with their hosts (Williamson and Gleason, 2003). However, both CNs and RKNs inject their salivary secretions in plant cells and withdraw nutrients (Atkinson et al., 1988; Li et al., 2011) by forming multi-cellular feeding sites called syncytia and giant cells, respectively. The infected plants remain stunted, show wilting symptoms and become prone to enhanced susceptibility to other diseases (Fairbairn et al., 2007). Migratory endoparasitic nematodes such as *Radopholus* spp. and *Pratylenchus* spp. are also highly damaging as in contrast to sedentary endoparasites, the migratory endoparasites remain motile and migrate through the course of their development.

Various methods have been used for management of PPNs including development of resistant cultivars, chemical nematicides and cultural practices. Use of nematicides as means of chemical control has been most effective in managing PPNs. However, detrimental effects of the major nematicides on environment and human health have compelled various developed and developing countries to impose bans on their use. Nematode resistant genes are present in some cultivars of species like tomato, potato and soybean but they are only limited to specific pathotypes while many crops do not have the presence of resistance loci (Fairbairn et al., 2007). This situation leads us to utilize technological advancements like RNA interference (RNAi) for engineering resistance against important nematode pests in crop plants. RNAi refers to sequence specific and homology-dependent gene silencing through a complex mechanism in which double stranded RNA (dsRNA) is recognized which leads to a chain of events resulting in the degradation of both the dsRNA and homologous RNA. Since its first description in *Caenorhabtidis elegans* (Fire et al., 1998), this highly conserved mechanism of RNAi has been demonstrated in various organisms belonging to different species across the animal and plant kingdoms (Jones et al., 2011). After the onset of RNAi by exogenous dsRNA, the intestinal epithelial cells of the nematodes are generally involved in dsRNA uptake. In case, there is no expression of the target gene in the uptake cells, silencing of the target transcript is systematically spread to more distant cells (Whangbo and Hunter, 2008). The most imperative aspect of RNA interference is that, it is highly precise, remarkably powerful and the interference can be caused in cells and tissues far away from the site of introduction (Rosso et al., 2009; Tomoyasu et al., 2008).

## RNAi in PPNs by soaking in dsRNA solutions

*In vitro* RNAi has been successfully demonstrated in CNs (Urwin et al., 2002), RKNs (Rosso et al., 2005) and even migratory nematodes (Haegeman et al., 2009; Soumi et al., 2012; Li et al., 2015) by feeding dsRNA solutions. Three different methods have been used in *C. elegans* for introduction of dsRNA which include feeding on bacteria expressing target gene dsRNA (Timmons and Fire, 1998; Kamath et al., 2001; Timmons et al., 2001), soaking of nematodes in dsRNA solution facilitating its oral uptake (Tabara et al., 1998) and microinjection (Fire et al., 1998; Mello and Conte, 2004). But, in case of PPNs, microinjection has not been effective because of the small size of the infective stages and their inability to ingest fluid without host plant infection. However, a variety of technical and chemical advances have successfully enhanced efficient dsRNA uptake from solutions by soaking. Urwin et al. (2002) first demonstrated *in vitro* RNAi in PPNs successfully by using a neuroactive compound, octopamine to facilitate dsRNA uptake by second stage juveniles (J2s) of CNs *H. glycines* and *G. palida*. In further studies, induction of dsRNA uptake by *Meloidogyne incognita* J2s was enhanced by using the same method (Bakhetia et al., 2005; Shingles et al., 2007). In other studies, resorcinol and serotonin were used for successful uptake of dsRNA in *M. incognita* (Rosso et al., 2005; Huang et al., 2006) and lipofectin was used in case of *Bursaphelenchus xylophilus* (Park et al., 2008). Fluoroscein isothiocyanate (FITC) have been used as a visual marker for monitoring dsRNA uptake and selection of individuals in various studies (Urwin et al., 2002; Rosso et al., 2005; Dutta et al., 2015b).

Different time periods of exposure of J2s to dsRNA in a range of 1 h to 7 days have been employed in different studies. For the knockdown of *Mi-gsts-1, M. incognita* J2s were incubated with *Mi-gsts-1* dsRNA for 1 h with 90% reduction in the transcript expression (Dubreuil et al., 2007). Bakhetia et al. (2005) demonstrated highly efficient *in vitro* dsRNA uptake by J2s of *M. incognita* with 4 h incubation period. However, further studies showed increase in transcript reduction resulting in desired phenotypic effects with increase in the incubation time (Kimber et al., 2007; Jones et al., 2011). Majority of the reports showing *in vitro* dsRNA soaking have not assessed the stability of the resulting gene knock down. However, the efficiency and duration of the silencing effect was assessed for *M. incognita* calreticulin (*Mi-crt*) and polygalacturonase (*Mi-pg-1*) by Rosso et al. (2005). The transcript repression was highest at 20 and 44 h after soaking into dsRNA solution for *Mi-crt* and *Mi-pg-1* respectively. However, the silencing effect was not detectable for both the genes 68 h after soaking. In *H. glycines*, the reduction in the transcript levels of β-1,4- endoglucanase was observed immediately after an incubation of 16 h with the corresponding dsRNA and after 15 days of dsRNA treatment, the transcript levels returned to normal (Bakhetia et al., 2007). These reports suggest that the silencing achieved due to soaking in dsRNA solutions is often transient and lack stability. In general, it can be concluded that the soaking method is an efficient tool for identification of gene function and expression. However, the obligate parasitic nature of plant parasitic nematodes and their exclusivity to feed on plant cells throughout their life cycle inside the host makes *in planta* RNAi technology a suitable approach to combat plant parasitic nematodes. The advantage of the host delivery strategy is that it provides a continuous availability of the dsRNA to the nematode, thereby making the chances of gene suppression reversal remote.

## Host generated RNAi to silence nematode specific genes

**Figure 1:**
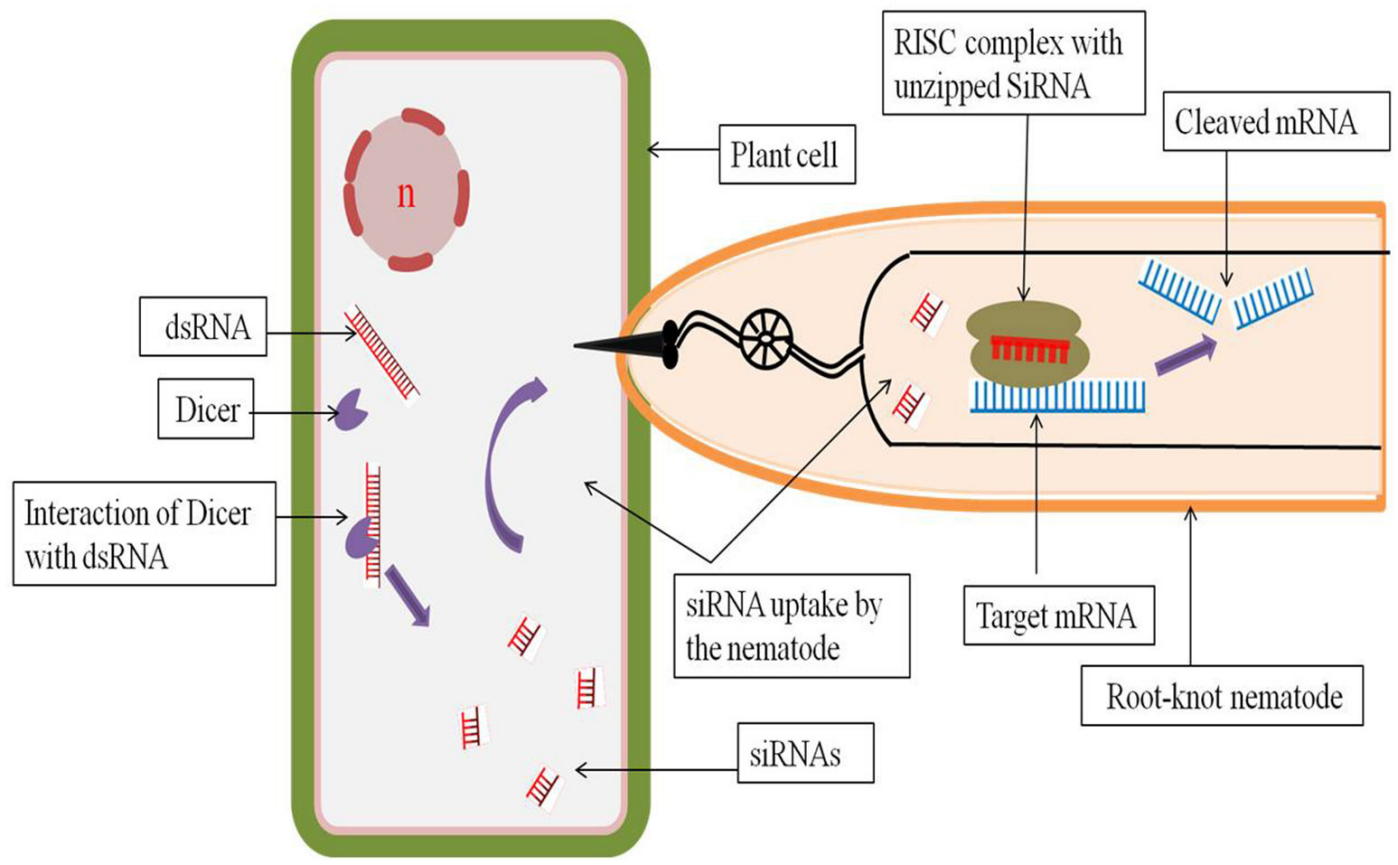
**Host generated RNAi through interaction between host plant cell and root-knot nematode**. The dsRNA introduced into the host plant is recognized by the cellular RNAse III type enzyme dicer, which cuts the dsRNA into shorter fragments of 20–25 nucleotides called siRNAs. During infection into the host, the nematode ingests the siRNAs through its stylet. These host derived siRNAs are then processed by the nematode RNAi machinery where the unzipped siRNAs bound to the RISC complex cleaves the target mRNA in a sequence specific manner and inhibits further translation of the target mRNA.

Host generated RNAi has proved to be a revolutionary approach for the delivery of dsRNAs or siRNAs into the feeding nematodes for the silencing of vital nematode specific genes. Purposely, those genes should be targeted whose expression is essential for the nematodes after the feeding starts to ensure a highly lethal phenotype. A dsRNA construct for the target gene is developed by cloning a part of the target gene cDNA in sense and antisense orientation separated by an intron or spacer region. A strong tissue specific or constitutive promoter may be used to drive the expression of the dsRNA. Transcription of the sense and antisense strands results in the formation of a self-complimentary hairpin structure with the removal of the intron by splicing (Smith et al., 2000). The dsRNAs so formed can either be directly ingested by the PPNs or can be processed by the host plant's own RNAi machinery and the resulting siRNAs can be subsequently ingested by the PPNs (Bakhetia et al., 2005; Dutta et al., 2015a; Figure 1).

Host generated RNAi has been demonstrated by targeting different nematode genes which may be broadly classified under three categories: housekeeping genes, parasitism or effector genes and genes associated with nematode development. Yadav et al. (2006) first demonstrated host delivered RNAi in tobacco to silence two nematode specific housekeeping genes; splicing factor and integrase of *M. incognita*. They reported more than 90% reduction in established nematodes in both the cases of host generated RNAi. Since then, host generated RNAi has been effectively demonstrated against PPNs by various groups of scientists all over the world (Table 1). Recently, Kumar et al. (2017) reconfirmed the utility of splicing factor and integrase as lethal RNAi targets for *M. incognita* by demonstrating significant reduction in number of galls, females and egg masses by targeting these genes for host generated RNAi in *A. thaliana*. Silencing of some of the other housekeeping genes like ribosomal protein 3a, ribosomal protein 4, spliceosomal SR protein, *Mi-Rpn7, Prp-17* etc. have resulted in substantial reduction in number and parasitism of incursive PPNs (Klink et al., 2009; Li et al., 2010b; Niu et al., 2012). In an innovative approach toward gene stacking, host generated RNAi was used to obtain combined resistance against migratory root lesion nematode, *Pratylenchus vulnus* and crown gall in walnut through co-transformation (Walawage et al., 2013). They used *A. tumefaciens* carrying self-complementary *iaaM* and *ipt* transgenes and *A. rhizogenes* carrying *Pv010* gene from *P. vulnus* for co-transformation. Most of the reports involving nematode housekeeping genes indicate their effectiveness as efficient targets for host generated RNAi. However, silencing of a putative transcription factor of *M. javanica, MjTis11* through host generated RNAi in tobacco did not result in lethal phenotypic effect on the nematodes (Fairbairn et al., 2007). siRNA generation was detected in the transgenic plants confirming the successful processing of the delivered dsRNAs. Therefore, it can be concluded that either this gene is not a suitable candidate for host generated RNAi or the achieved downregulation levels of the transcript is not sufficient to generate lethal phenotypic effects. The unsuitability of genes as targets for host generated RNAi can possibly result from the genetic redundancy of such genes.

**Table 1:** **Host generated RNAi in plant parasitic nematodes**.

**Target gene**	**Nematode species**	**Host plant**	**promoter**	**phenotype**	**Time point**	**References**
Integrase	*M. incognita*	Tobacco	35S	>90% reduction in number of established nematodes	6–7 weeks after infection	Yadav et al., 2006
Splicing factor	*M. incognita*	Tobacco	35S	>90% reduction in number of established nematodes	6–7 weeks after infection	Yadav et al., 2006
Secreted peptide *16D10*	*M. incognita*	Arabidopsis	35S	63–90% reduction in number of galls and gall size	4 weeks after infection	Huang et al., 2006
	*M. arenaria*					
	*M. javanica*					
	*M. hapla*					
Major sperm protein	*H. glycines*	Soybean	ACT2	Upto 68% reduction in number of eggs	8 weeks after infection	Steeves et al., 2006
Putative transcription factor	*M. javanica*	Tobacco	35S	None	6 weeks after infection	Fairbairn et al., 2007
Ribosomal protein 3a	*H. glycines*	Soybean	FMV-sgt	87% reduction in number of female cysts	8 days after infection	Klink et al., 2009
Ribosomal protein 4	*H. glycines*	Soybean	FMV-sgt	81% reduction in number of female cysts	8 days after infection	Klink et al., 2009
Spliceosomal SR protein	*H. glycines*	Soybean	FMV-sgt	88% reduction in number of female cysts	8 days after infection	Klink et al., 2009
*4G06*, Ubiquitin-like	*H. schachtii*	Arabidopsis	35S	23–64% reduction in number of developing females	14 days after infection	Sindhu et al., 2009
*3B05*, cellulose binding protein	*H. schachtii*	Arabidopsis	35S	12–47% reduction in number of developing females	14 days after infection	Sindhu et al., 2009
*8H07*, SKP1-like	*H. schachtii*	Arabidopsis	35S	>50% reduction in number of developing females	14 days after infection	Sindhu et al., 2009
*10AO6*, Zinc finger protein	*H. schachtii*	Arabidopsis	35S	42% reduction in number of developing females	14 days after infection	Sindhu et al., 2009
*Y25*, beta subunit of COPI complex	*H. glycines*	Soybean	35S	81% reduction in number of nematode eggs	5 weeks after infection	Li et al., 2010a,b
*Prp-17*, Pre-mRNA splicing factor	*H. glycines*	Soybean	35S	79% reduction in number of nematode eggs	5 weeks after infection	Li et al., 2010b
*Cpn-1*	*H. glycines*	Soybean	35S	95% reduction in number of nematode eggs	5 weeks after infection	Li et al., 2010b
*Rpn7*	*M. incognita*	Tomato	35S	Reduction in motility and infectivity of J2s	40 days after infection	Niu et al., 2012
*AF531170*, parasitism gene	*M. incognita*	Tomato	35S	54–59% reduction in number of developing females	7, 21, 30 days after infection	Choudhary et al., 2012
*8D05*, Parasitism gene	*M. incognita*	Arabidopsis	35S	Reduction in number of galls	8 weeks after infection	Xue et al., 2013
*flp-14*, FMRF amide like peptide	*M. incognita*	Tobacco	35S	Reduction in parasitic ability from 67–86%	30 days after infection	Papolu et al., 2013
*flp-18*, FMRF amide like peptide	*M. incognita*	Tobacco	35S	Reduction in parasitic ability from 53–82%	30 days after infection	Papolu et al., 2013
*Mi-ser-1*, serine protease	*M. incognita*	Tobacco	35S	Reduction in number of eggs per gram of root. Reduction in egg hatching ratio	28 days after infection	Antonino de Souza Júnior et al., 2013
*Mi-cpl-1*, Cysteine protease	*M. incognita*	Tobacco	35S	Reduction in number of eggs per gram of root.	28 days after infection	Antonino de Souza Júnior et al., 2013
*Mi-asp-1*+*Mi-ser-1*+*Mi-cpl-1* (fusion)	*M. incognita*	Tobacco	35S	Reduction in number of eggs per gram of root.	28 days after infection	Antonino de Souza Júnior et al., 2013
*Pv010*	*P. vulnus*	Walnut	35S	Reduction in number of nematodes	60 days after infection	Walawage et al., 2013
*Mc16D10L*	*M. chitwoodi*	Potato	35S	65–68% Reduction in the number of egg masses	35, 55 days after infection	Dinh et al., 2014b
*Mc16D10L*	*M. chitwoodi*	Arabidopsis	35S	57 and 67% reduction in number of egg masses and eggs respectively	35, 55 days after infection	Dinh et al., 2014a
*Mi-cpl-1*	*M. incognita*	Tomato	35S	60–80% reduction in infection and multiplication	35 days after infection	Dutta et al., 2015b
*Pp-pat-10*	*P. penetrans*	Soybean	35S	Upto 40% reduction in number of nematodes	3 months after infection	Vieira et al., 2015
*Pp-unc-87*	*P. penetrans*	Soybean	35S	Upto 50% reduction in number of nematodes	3 months after infection	Vieira et al., 2015
*Rs-cb-1*	*R. similis*	Tobacco	35S	Reduced reproduction and pathogenicity	75 days after infection	Li et al., 2015
*HSP90, Heat Shock Protein*	*M. incognita*	Tobacco	35S	Delayed gall formation and upto 46% reduction in the number of eggs		Lourenço-Tessutti et al., 2015
*ICL, Isocitrate lyase*	*M. incognita*	Tobacco	35S	Upto 77% reduction in egg oviposition		Lourenço-Tessutti et al., 2015
*Unc-15*	*Ditylenchus destructor*	Sweet potato	35S	50% reduction in the infection area	45 days after infection	Fan et al., 2015
*MeTCTP*	*M. enterolobii*	Tomato	35S	Reduction in number of nematodes	15 and 30 days after infection	Zhuo et al., 2017
*MiMSP40*	*M. incognita*	Arabidopsis	35S	Reduction in the number of galls	7 weeks after infection	Niu et al., 2016

Both, *in vitro* and *in vivo* RNAi approaches were used to silence a parasitism gene, *16D10*, expressed in the subventral gland cells of *M. incognita* leading to substantial reduction in the number of galls in the range of 63–90% in *Arabidopsis* (Huang et al., 2006). In addition, the gall size also decreased leading to reduction in the total number of eggs as compared to control. *Mc16D10L* was also targeted for host delivered RNAi in potato and *Arabidopsis* against *M. chitwoodi* by Dinh et al. (2014a,b) leading to significant reduction in nematode numbers in terms of the number of eggs and egg masses. They also reported substantial reduction in target gene expression in the second generation *M. chitwoodi* eggs and J2s which demonstrates transmission of the RNAi effect into the progenies. *16D10* is conserved among the *Meloidogyne* spp. which further makes it a suitable target for engineering resistance against a broader range of PPNs. Parasitism of PPNs was successfully suppressed by targeting some other parasitism or effector genes like *4G06, 3B05, 8H07, 10AO6, AF531170, 8D05, MeTCTP etc. through host generated RNAi* (Sindhu et al., 2009; Choudhary et al., 2012; Xue et al., 2013; Zhuo et al., 2017). Recently, *M. incognita* effector *MiMSP40* was reported to facilitate parasitism through manipulation of plant immunity (Niu et al., 2016). Overexpression of *MiMSP40* lead to increased susceptibility in *Arabidopsis*, while host generated RNAi of *MiMSP40* resulted in reduction in parasitism and reproductive potential of *M. incognita*. Similarly, overexpression of *M. enterolobii* effector *MeTCTP* imparted increased susceptibility. Conversely, *in planta* RNAi targeting *MeTCTP* caused attenuated parasitism in *Arabidopsis* (Zhuo et al., 2017). Therefore, recent reports indicate the suitability of nematode effector genes as targets for host generated RNAi to achieve resistance.

Genes involved in nematode development and reproduction are often targeted with considerable success in hindering the development and reproductive potential of PPNs (Steeves et al., 2006; Charlton et al., 2010; Antonino de Souza Júnior et al., 2013; Papolu et al., 2013; Dutta et al., 2015b). Decreased number of eggs was phenotypically observed by targeting a major sperm protein from *H. glycines* for host generated silencing in soybean (Steeves et al., 2006). Silencing of the major sperm protein hampered the reproductive potential of *H. glycines* which continued to the next generation of the nematodes as the progeny also showed an impaired ability to reproduce successfully. FMRF amide like neuropeptides were targeted for silencing through *in vitro* and *in vivo* RNAi by Papolu et al. (2013). Transgenic tobacco lines expressing flp*-14* and *flp-18* dsRNAs showed significant reduction in the range of 50–80% in the infection and multiplication of *M. incognita*. This study proved that neuropeptides can be exploited as potential targets for host delivered RNAi considering the involvement of these genes in nematode physiology including locomotion, feeding, parasitism and reproduction. *In vitro* silencing of carrying cathepsin L cysteine proteinase (*Mi-cpl-1*), led to reduced attraction and penetration of *M. incognita* in tomato suggesting its role in nematode parasitism (Dutta et al., 2015b).

The development of dsRNA constructs for host delivered RNAi through conventional methods have been a time taking and tedious. Therefore, a much easier, quick and effective process of gateway cloning system has been employed by different groups for the development of RNAi constructs (Klink et al., 2009; Papolu et al., 2013; Dutta et al., 2015b). *A. rhizogenes* mediated hairy root method for transformation in crops like tomato (Remeeus et al., 1998), soybean (Klink et al., 2009; Li et al., 2010b) and sugar beet (Kifle et al., 1999; Cai et al., 2003) has been instrumental for rapid screening of the target genes. The genes found effective through hairy root transformation can further be utilized for the development of stable transgenic plants through *A. tumifeciens* mediated transformation. The studies on host delivered RNAi have showed that the success in obtaining significant degree of resistance depends on the role of the target gene and the degree of transcript knockdown. However, of all the strategies implied till date to achieve resistance against PPNs, host-delivered RNAi appears to provide more effective resistance when it targets nematode genes involved in essential cellular processes.

## Biosafety aspects

Application of RNAi for management of PPNs needs thorough risk assessment and proper designing of the experiments to overcome the limitations. Avoiding off-target effects is an important consideration for all RNAi experiments. Off target effects can originate due to sequence identity between dsRNA and non target mRNA transcripts resulting in a compromised specificity of RNAi and false interpretation of the resulting phenotype. According to Rual et al. (2007); in *C. elegans*, a sequence of mRNA having more than 95% identity with the dsRNA for over 40 nucleotides results in off-target effects. Unexpected and unintended gene silencing in the plant may lead to harmful and deleterious effects on its phenotype and physiology and can cause serious environmental consequences. Xing and Zachgo (2007) explained the phenomenon of pollen lethality in *Arabidopsis* where surprising pleiotropic effects such as reduced pollen viability was observed due to RNAi, however other plant growth parameters were found to be normal. Apart from these off target effects, non-target organisms may encounter harmful non target effects of RNAi on being exposed to the plant parts or debris of genetically modified plants through RNAi. Therefore, development of strategies for preventing the off-target and non-target effects is a crucial biosafety consideration for a wider employment of RNAi as a novel tool in plant disease management.

Availability of suitable genomic databases can be utilized extensively for *in silico* homology searches for selection of target genes to avoid off-target effects (Banerjee et al., 2017). Genes showing high degree of sequence conservation among plant and animal kingdom should be avoided and use of species specific targets should be encouraged. The 5′-3′ untranslated regions (UTR) sequences can also be used as siRNA targets owing to their less degree of conservation as compared to coding regions. Thorat et al. (2016) used a nematode responsive and root specific promoter of *Arabidopsis* origin to transform tomato with a GUS reporter gene. A strong GUS activity was reported at nematode infection site starting from 10 days up to 21 days post infection. Further, this promoter was used to drive the expression of *M. incognita* splicing factor dsRNA and upon transformation in tomato; 50–70% reduction in nematode galls over the control plant was reported. This strategy of using root specific and/or nematode inducible promoters can avoid the expression of siRNAs in undesirable parts of the plants.

## Conclusion and future prospects

RNAi has emerged as a powerful strategy to control multiple pest and pathogens including nematodes especially as we are moving toward the goal of phasing out chemicals that are harmful to environments and ecosystems. Management of nematodes however, presents some unique challenges as these are obligatory parasites requiring living host for feeding. The use of host induced RNAi to combat plant pathogenic nematodes has so far been effective especially with respect to RKNs and CNs. The advancement in the area of functional genomics availability of genome sequence data and new bioinformatics tools have enabled design and engineering of effective dsRNA expression constructs addressing concerns of off-target silencing. Stacking of dsRNA sequences to target multiple genes has emerged as an attractive proposition for effective nematode control. Use of nematode induced and plant tissue specific promoters limiting dsRNA gene expressions to specific plant tissue/s in response to particular nematode can also mitigate biosafety concerns. The ability to precisely edit genomes is rapidly transforming the landscape of novel ways to target plant pathogens. CRISPR/Cas system is emerging as a powerful approach for loss of function analysis, insights into host–parasite and parasite–vector interactions, and the genetic basis of parasitism. A number of CRISPR/Cas9 genome editing protocols have been established in *C. elegans* (Friedland et al., 2013) opening new doors to studying the biology of closely related nematode parasites. Translation of CRISPR/Cas9 technology from *C. elegans* to *Strongyloides spp*., *Ascaris suum, Brugia malayi* and *Haemonchus contortus* have been recently outlined (Ward, 2015; Britton et al., 2016; Zamanian and Andersen, 2016). Recent reports on topical application of dsRNA for resistance against viruses using layered double hydroxide clay nanosheets (Mitter et al., 2017) opens up possibilities to exploit such innovations for specific and combinatorial resistance against PPNs, insects and plant pathogenic fungi.

## Author contributions

SB, AB, SG, OG, and AS drafted the manuscript. SB and AB collected background information. SG, PJ, AD, and AS critically revised the manuscript. All authors read and approved the final manuscript.

### Conflict of interest statement

The authors declare that the research was conducted in the absence of any commercial or financial relationships that could be construed as a potential conflict of interest.
